# Effective long-term control of *Echinococcus multilocularis* in a mixed rural-urban area in southern Germany

**DOI:** 10.1371/journal.pone.0214993

**Published:** 2019-04-12

**Authors:** Andreas König, Thomas Romig, Ernst Holzhofer

**Affiliations:** 1 Technical University of Munich, School of Life Science Weihenstephan, Wildlife Biology and Management, Freising, Germany; 2 University of Hohenheim, Parasitology Unit, Stuttgart, Germany; 3 Holzhofer Flight Service, Boxberg, Germany; Justus-Liebeig University Giessen, GERMANY

## Abstract

Effective preventive strategies are available to control *Echinococcus multilocularis* in foxes in order to reduce the human infection risk. Reduction of *E*. *multilocularis* prevalence in foxes was achieved in various studies by distributing praziquantel-containing bait by hand or by aircraft in either rural or settlement areas. Here, an integrated approach is described from southern Germany (district of Starnberg). Baseline data were obtained in winter 2002/03, when the prevalence rate in the project area was 51%. Between December 2005 and December 2011, air distribution of bait in agricultural and recreational areas was combined with distribution by hand in towns and villages, in order to cover the entire fox population, with a bait density of 50 pieces / km^2^ (baiting area: 213 km^2^). In addition, a control area without anthelmintic treatment was selected. Prevalence was reduced in the baiting area to 1% by March 2007. Subsequently, from 2007 to the end of 2011, prevalence rates remained at a low level with 2.4% (2007), 2.4% (2008), 2.6% (2009), 1.2% (2010) and 0.0% (2011). In the un-baited control area the prevalence rates remained high, ranging from 19.6% to 35.1% with an average of 27.3%. During the 6 years of anthelmintic treatment, differences between baiting and control areas were highly significant (*P*<0.001). In the suburban and urban parts of the study area prevalence could be reduced to less than 1%, i.e. to a level below the limit of detection, which was maintained even after the measures had been discontinued. The applicability and effectiveness of anthelmintic baiting was therefore confirmed even for a heavily settled and fragmented landscape, which posed challenges for practical application of the control measures. The cost of the project ranged between € 1.70 and € 2.00 per inhabitant of the baiting area per year.

## Introduction

*Echinococcus multilocularis* is a zoonotic cestode of foxes and other canids, whose metacestode utilises rodents and other small mammals as intermediate hosts. Accidental infection of humans leads to alveolar echinococcosis (AE), which is potentially fatal due to the progressing occupation of space by the metacestode, analogous to a malignant tumour [1]. *E*. *multilocularis* and AE are widely distributed across temperate and cold regions of the northern hemisphere; in Europe, the parasite is present in most regions except southern Europe, the British Isles and most parts of Scandinavia [2]. There was a drastic emergence of this parasite in central Europe and elsewhere at the end of the 20^th^ century, in conjunction with a several-fold increase in fox populations [3], a marked tendency of foxes to establish populations within human settlements, including cities, and an increased parasite transmission within human settlements [4, 5, 6, 7, 8, 9,10]. The publicly perceived increase in the human infection risk prompted various studies on the feasibility of controlling the parasite through the anthelmintic treatment of foxes with baits, using a variety of different bait types, baiting schedules (frequency, bait density), landscapes (rural, urban) and area sizes [11, 12, 13]. Consistent outcomes of the various approaches were (a) the effectiveness in decreasing *E*. *multilocularis* prevalence in foxes in different environments, in different regions and with very different sizes of study areas (<10 to >4500 km^2^) [12], (b) the need for long-term application of the methods and (c) the failure to completely eliminate parasite transmission during baiting periods ranging from 9 months to 6 years [12, 14]. Persistence at low level was explained by small study areas (facilitating immigration of non-treated foxes), exclusive reliance on bait distribution by aircraft, which allows transmission to persist in and around villages and towns, inadequate bait densities in relation to rising fox populations, and short application periods.

Densely settled areas are typical for central Europe, where larger cities, suburban areas, towns and highly fragmented agricultural lands merge into loose conurbations, so that it is difficult to differentiate between ‘urban’ and ‘rural’ areas. Specialised populations of foxes in Europe have successfully adapted to such anthropogenic landscapes, reaching population densities that exceed those in natural or sparsely settled rural areas by far. Wherever suitable habitats for intermediate hosts (mainly common voles) persist in such conurbations, *E*. *multilocularis* is able to complete its life cycle. Lower prevalence rates in foxes are offset by higher fox densities, which leads to potentially higher infection risks for humans due to frequent human-fox contact [6, 15, 16, 17]. For south-eastern Germany (Bavaria), it has been estimated that the probability of human contact with infectious fox faeces is 45 times higher in the city of Munich than on average in Bavaria [18].

As the number of human AE cases are on the increase in central Europe [19, 20] and no effective measures can be proposed to minimize individual human infection risk, interruption of the lifecycle through anthelmintic baiting appears to be the best preventive measure. In terms of cost-benefit calculations, baiting in urban or suburban areas is considered far more cost effective than in rural landscapes [12]. Baiting in small-scale urban areas has been shown to be effective even in the long term [21, 22].

In mixed urban-rural landscapes, which are typical for large parts of central Europe, the application of anthelmintic fox baiting poses challenges, due to highly fragmented landscapes that require different and flexible methods of bait distribution. Here, a study of anthelmintic baiting was conducted in a mixed rural-urban conurbation in the vicinity of Munich (Bavaria, Germany) using an integrated approach of bait distribution by air and by hand.

## Methods

The study area and methods are to a large degree identical to those described for the initial phase of the project [23]. In the following we present an abbreviated version, highlighting differences to the previous approach.

### Study area

**Fig 1:**
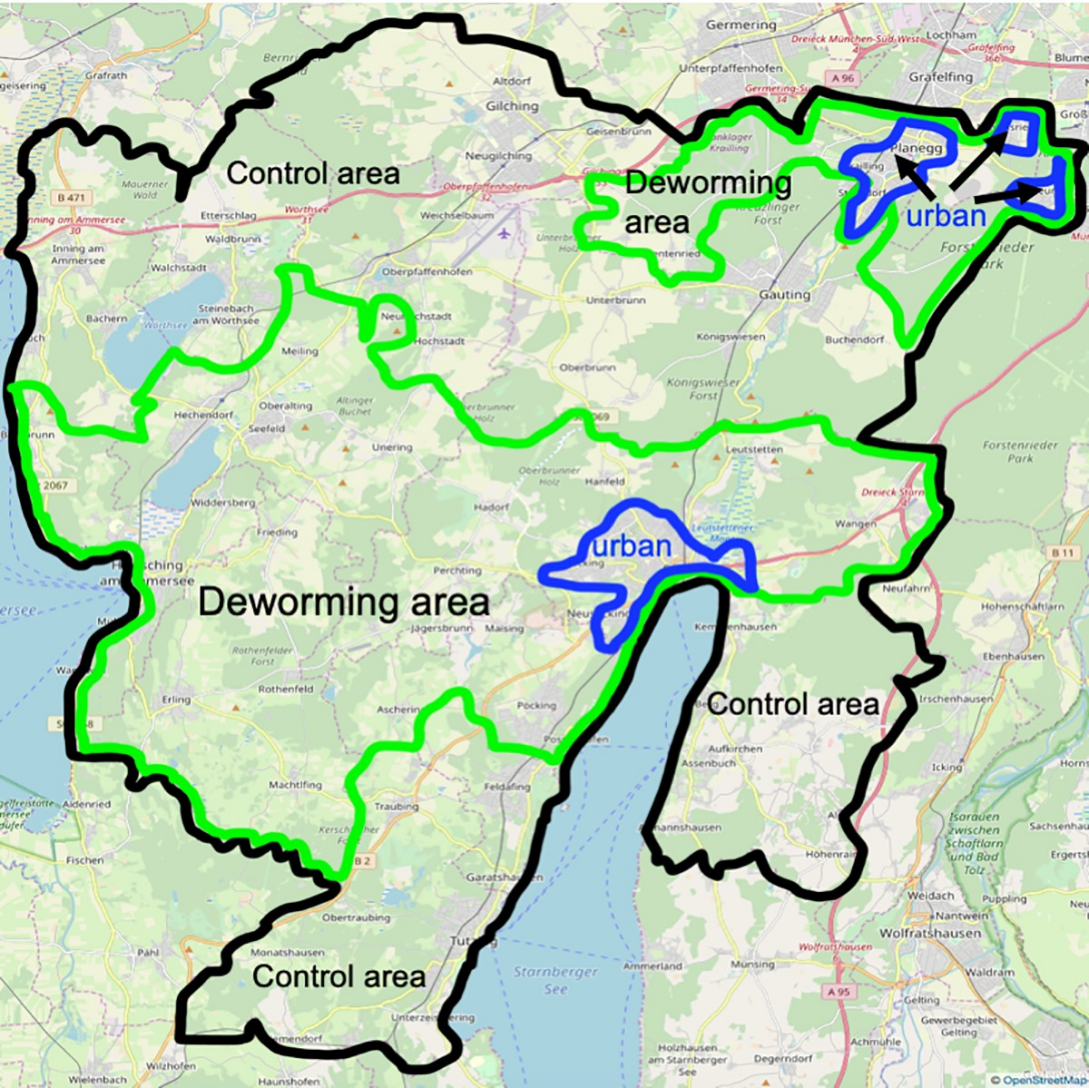
Project area. **Black line project area, green line deworming area and blue line suburban / urban area with urban fox populations.** This Fig 1 is made available under the Open Database License: http://opendatacommons.org/licenses/odbl/1.0/. Any rights in individual contents of the database are licensed under the Database Contents License: http://opendatacommons.org/licenses/dbcl/1.0/.

The total area comprises 488 km^2^, and is situated in the pre-alpine lake region of the German federal state of Bavaria (Fig 1). It is characterized by two large lakes (Lakes Starnberg and Ammer), inter-connecting large and small river and brook systems, and natural or artificial ponds. The landscape is highly anthropogenic and fragmented, consisting mainly of pastures and meadows interspersed with human settlements of different sizes. The climate is influenced by the vicinity of the Alps. The average annual temperature is 7^0^ C, and annual precipitation is well above 1000 mm [7].

Towns and villages in the study area are known to support fox populations with various degrees of adaptation to urban environments (‘village foxes’, ‘urban foxes’) [24, 25]. Towns and villages with more than 10000 inhabitants in a connected settlement area were defined for the purposes of the analysis as urban areas. Some urban fox populations have little contact with rural fox populations and little access to rodents in rural areas [18, 26, 27]. These inhabit the settlement area of the town of Starnberg and the villages Krailling, Planegg and Neuried.

Anthelmintic baits were distributed over an area of 213 km^2^, with the remaining area of 275 km^2^ serving as a control area.

### Collection of fox samples

To monitor the change of prevalence over time, foxes shot in the course of traditional hunting were collected. Local hunters deposited fox carcasses in plastic bags labelled with the date, location and hunter’s name in project deep-freezers. Foxes were not collected in the months February to July of each year. Fox carcasses were stored at -15 to -20°C for a maximum of 3 months and subsequently transported in frozen condition to the diagnostic laboratory.

During this project phase, a total of 1575 foxes were collected and examined (1058 from the baiting area including 266 from the suburban / urban area, 517 from the control area).

### Echinococcus multilocularis detection

After thawing for approximately 36 hours, the small intestines were removed using double ligatures and opened longitudinally. Worm detection was carried out using the ‘intestinal scraping technique’ (IST) as described earlier [28]. Compared to the ‘‘sedimentation and counting technique’ (SCT) as a gold standard, the method was estimated to give a sensitivity of 78% under conditions of high endemicity, while retaining 100% specificity [5].

### Baiting strategy

Anthelmintic baits with a matrix of Altrofox 91 containing 50 mg of Praziquantel (Bayer AG) were distributed at densities of 50 baits/km^2^ every 4 weeks in 2006, and every 6 weeks in the years 2007 to 2009. In 2010 and 2011, the frequency of distribution was reduced to 5 times per year.

Baits were distributed in rural and suburban/ urban areas using different methods.

#### Rural area

Bait distribution was done by aircraft as described earlier [23], over forests, open countryside, meadows, farmland and wetlands. The distance between flight lines was 1 km (2005–2008) and 500 m (2009–2011). In addition, bait was also distributed by hand around fox dens in agricultural and recreational areas during the mating and cub-rearing seasons (January / February and June / July).

#### Suburban and urban area

As distribution by aeroplane over urban areas is legally not permitted in Germany, bait was therefore distributed by hand and mainly placed in house gardens [23]. In suitable places, an uptake rate of up to 90% (average 75%) was achieved within 2–3 days [29]. Distribution points within gardens or public areas were selected according to published criteria [30]. The gardens were selected based on observations by garden owners, or according to the “fox garden model” [27]. This ensured that the baits were predominantly taken by foxes.

### Ethics statement and permissions

All hunted animals were collected in accordance with German Hunting Regulations, during the legal hunting season, and as part of legal hunting activities. Foxes were hunted and collected by local hunters, who either held local hunting permits or were the owners of hunting rights for the area. No additional approval by an animal research ethics committee is necessary for legally hunted animals.

The permission to hunt in suburban areas was given by the regional hunting authorities of the districts of Starnberg and Munich. If foxes were hunted on private land, the owners were asked for permission.

The Government of Upper Bavaria was informed by the bait producer (Bayer AG) of the clinical field study of the baiting and deworming of foxes. Further permits were not required.

### Statistics

The confidence intervals [31] and T-tests for equal and unequal variances were used to back up the statistical differences. The decision on whether to use a t-test for equal or unequal variances was taken using *Levene’s Test for Equality of Variances*. The calculations were carried out using SPSS 24.0.

## Results

**Fig 2:**
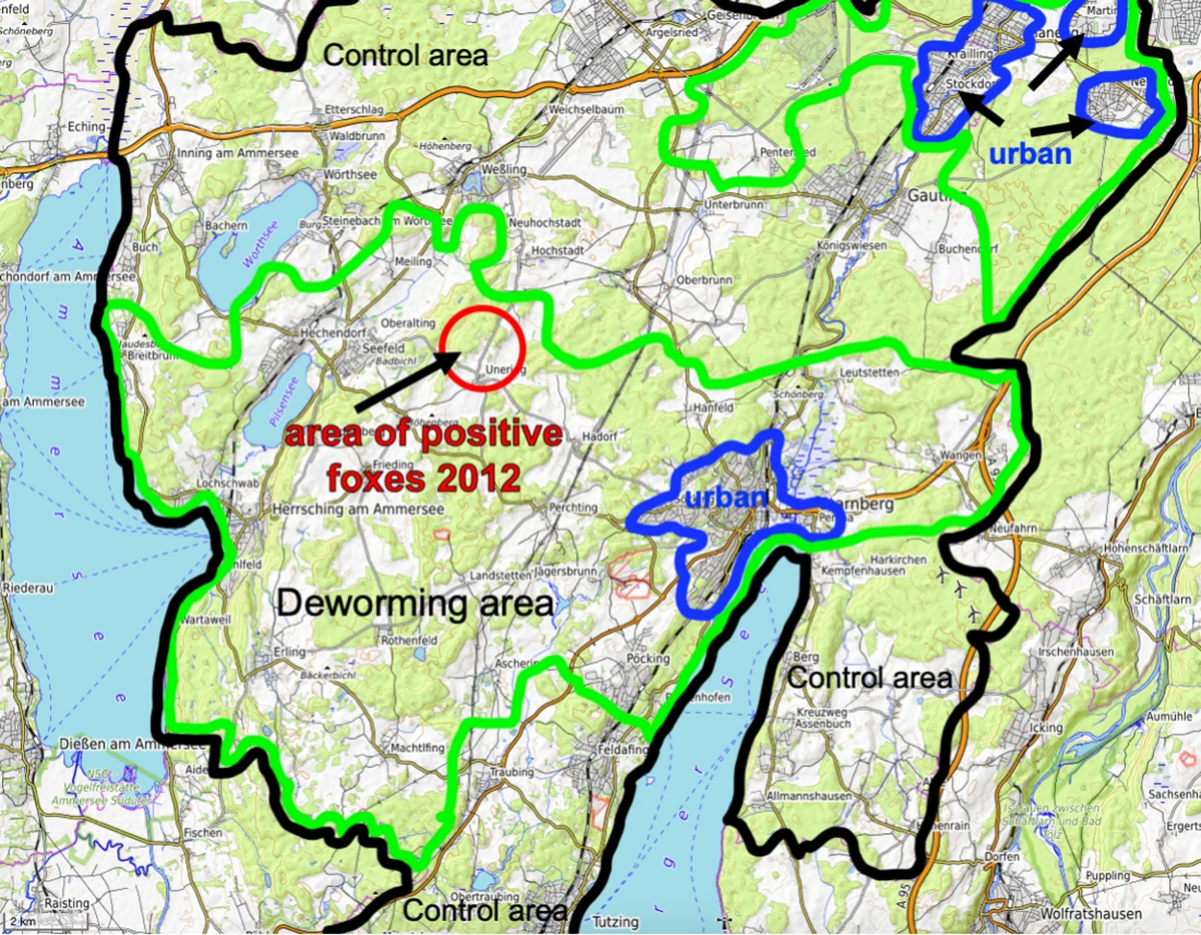
Project area with area of positive foxes after deworming in 2012. Entire project area (black line), deworming area (green line) and suburban / urban areas with urban fox populations (blue line). This Fig 2 is made available under the Open Database License: http://opendatacommons.org/licenses/odbl/1.0/. Any rights in individual contents of the database are licensed under the Database Contents License: http://opendatacommons.org/licenses/dbcl/1.0/.

The change of prevalence over time is shown in Table 1. Immediately prior to the baiting period, prevalence was 37.5% (baiting area) and 39.0% (control area). During the baiting phase, the prevalence in the baiting areas was rapidly reduced to 5.0% during 2006, and continued to decrease towards 0.0% in 2011. Importantly, prevalence did not rise after the increase in the length of intervals between bait distributions from 1 to 2.5 months after 2009. After baiting was stopped in December 2011, however, prevalence increased to 10.7% in the following year, although positive foxes were found on the edges of the baiting area (Fig 2).

**Table 1:** Change of prevalence over time in the deworming and control area (confidence interval 95% both sides [31], *N* = *1575*).

		Baiting area				Control area			
**Period**	**Baiting intervall**	**no. examined / positive**	**% positive**	**CI—95%**	**CI + 95%**	**no. examined / positive**	**% positive**	**CI—95%**	**CI + 95%**
2002/2003	No baiting	112/ 58	51.8	41.6	61.8	164 / 85	51.8	43.9	59.5
2005	No baiting Jan—Nov	56 / 21	37.5	24.3	51.0	41 / 16	39.0	24.0	55.5
2006	4 week	160 / 8	5.0	2.0	10.0	77 / 27	35.1	25.1	46.1
2007	6 week	169 / 4	2.4	0.9	5.4	50 / 11	22.0	11.5	36.0
2008	6 week	125 / 3	2.4	0.7	5.9	34 / 12	35.3	19.8	52.3
2009	6 week	152 / 4	2.6	0.1	6.6	63 / 15	23.8	13.8	35.8
2010	10 week	165 / 2	1.2	0.0	3.5	51 / 10	19.6	9.6	33,4
2011	10 week	88 / 0	0	0.0	4.0	22 / 6	27.3	10.3	45.8
2012	No baiting	28 / 3	10.7	3.8	26.8	15 / 6	40.0	28.5	64.0
Sum	Baiting period	859 / 21	2.4	1.6	3.4	297 / 81	27.3	21.3	33.8

By contrast, the prevalence in the reference area remained between 19.6% and 40.0% during the entire project period. For 2006 to 2011, the mean prevalence of *E*. *multilocularis* in foxes in the baiting and reference areas differed significantly (*T* = 9.398, *DF* = 320.876, *P*<0.001 (95% on both sides), *Levene Test* ***F*** = 1010.296 *P* < 0.001). The corresponding confidence intervals do not overlap.

If only the data from the 192 suburban/urban parts of the project area are considered, the prevalence of 25,0% (immediately prior to the anthelmintic treatment) decreased to 3.7. in 2006, whereas no infected fox was detected from 2007 to 2011. After baiting was stopped in December 2011 prevalence rate remained at 0% (0% - 17% *CI* 95%; *N = 7*) **Table 2**)

**Table 2:** Prevalence rate over time in suburban / urban areas before deworming and during deworming (P = (confidence interval 95% both sides [31], *N = 266*).

Period	Baiting intervall	no. examined / positive	% positive	CI—95%	CI + 95%
2002 / 2003	No baiting	58 / 15	25.9	15.4	38.9
2005	No baiting Jan—Nov	8 / 2	25.0		
2006	4 week	27 / 1	3,7	0.2	18.2
2007	6 week	37 / 0	0	0	9.1
2008	6 week	27 / 0	0	0	11.6
2009	6 week	26 / 0	0	0	11.6
2010	10 week	37 / 0	0	0	9.1
2011	10 week	38 / 0	0	0	9.1
2012	No baiting	7 / 0	0		
Sum	**Baiting period**	192 / 1	0.5	0	2.5

## Discussion

In this study we confirmed the effectiveness of anthelmintic praziquantel treatment of foxes in a mixed urban-rural area of southern Germany. Prevalence could be rapidly reduced in the initial phase of the baiting period, and after 6 years of the treatment programme, the infection rate was close to 0% (CI 0%-4%). This is in accordance with simulations carried out by previous authors [32], which, starting with an initial prevalence of 55%, calculated a prevalence of 1% after 5 years of treatment. In the suburban / urban areas the prevalence rate of *E*. *multilocularis* could be sustained at 0% one year after treatment. In contrast to a previous simulation [32], however, the lack of infected animals could be sustained over the 6 year period. This result suggests that, provided a treatment area can be demarcated in accordance with the principles of wildlife biology, an elimination of *E*. *multilocularis* in foxes is possible even in larger areas. Possibly, by implementing measures only on the edges of the area, re-intruduction of the parasite from outside could be prevented.

We did not observe any increase of prevalence after the reduction in the frequency of distribution to 5 times per year as it had been observed in an earlier study [24]. Rather, the prevalence continued to decrease to a level of 0.0% in 2011.We can thus assume that we succeeded to interrupt the infection cycle in the core area (including the urban areas), and that the 2–4 *E*. *multilocularis* positive foxes found per year had migrated into the area from outside. This hypothesis is supported by the fact that once the deworming period had finished, positive foxes were still restricted to the edge of the deworming area (Fig 2).

All these animals fell under the speed of spread band of 0.3–3.16 km yr^-1^, as simulated previously [32]. In the suburban-urban areas, the infection rate remained at 0% after the end of the deworming programme. Unlike the rate in the deworming area, the prevalence of *E*. *multilocularis* in foxes remained as high in the reference areas as at the beginning of the study, although fluctuating considerably between years.

A prevalence reduction towards the detection threshold has not been achieved in previous studies. This is likely due to a combination of factors:

- High initial baiting frequency: unlike in several other baiting projects [24], bait was first distributed at 4-week intervals, and distribution was then maintained at 6-week intervals over a prolonged period (3 years).
- High bait density: as in the experiments in Zurich [21], 50 pieces of bait/km^2^ were distributed. In earlier projects in the Swabian Jura region on the other hand, baiting density was only 20 baits/km^2^ [24].
- Integrated approach: distribution by hand in suburban and urban areas was combined with distribution by aircraft outside the settlements. This ensured that a maximum proportion of the foxes from different habitats had access to baits.
- Improved distribution method: with respect to aircraft distribution, optimal coverage of the deworming area with bait was ensured by a distance between flight lines of only 500 m, flown north-south and east-west and combined with diagonal flight lines.
- Consideration of fox biology: bait was distributed during the mating season and during the cub-rearing season around the fox dens.

Looking at the suburban/urban areas, we can see that here, except during the transitional first 6 months of the deworming period, no further *E*. *multilocularis-* positive foxes were found. This suggests than complete elimination of *E*. *multilocularis* from heavily settled areas is possible. This may be facilitated by the population structure of ‘urban’ foxes, which are resident, stable and self-reproducing with very limited exchange with the more rural populations [33]. With the help of the habitat model [27], it is possible to ensure the foxes are well provided with bait. The effectiveness of this model was reflected by the very high rate of bait acceptance, where bait disappeared within three to four days [29]. Furthermore, rodents as intermediate hosts play a less important role for the nutrition of foxes in urban areas as in rural areas, limiting the extent of re-infection [34]. However, edge effects are also observable in the suburban/urban area, as shown by studies in Switzerland [21]. There it was possible to reduce an initially high prevalence to 5.5% by distributing 50 pieces of bait /km^2^ monthly over an area of 1 km^2^ in the suburban area of the city of Zurich. In an experimental area covering 6 km^2^, however, a prevalence reduction to 1.8% was achieved.

Our project area, with only 213 km^2^, was relatively small in comparison with projects in Baden-Württemberg [24, 35] and Brandenburg [36]. In a larger study area, the effectiveness of the applied measures could possibly have been better, as edge effects caused by immigrating foxes in September and during the mating season would have played a lesser role.

As deworming of wild foxes does not only affect *Echinococcus*, but also other non-zoonotic cestodes and trematodes, the question arises as to whether such a level of intervention in the ecosystem is justifiable. Given the current rise in the incidence of infection in the human population, particularly in southern-central Europe, the deworming program appears justified until other forms of disease prevention in humans (e.g. vaccines) are available. Deworming seems to be the only available tool to achieve reduction of infection pressure to humans, as intensive fox culling leads to an increase in the *E*. *multilocularis* prevalence in foxes [37].

Reduction of infection risk for humans in the project area can be approximately calculated [18]. After the anthelmintic treatment, the risk of contact with *E*. *multilocularis* eggs in the deworming area sank by 89% in the entire deworming area, and in the suburban / urban part of it even by 100% compared to the average across the state of Bavaria.

In addition to the scientific result, fox deworming met with a high level of acceptance by the general public. This was shown in surveys among the residents of a community in the southern outskirts of Munich, where anthelmintic baiting has been carried out on a small scale since 2001 [38].

The costs of the measures, which range between € 1.70 and € 2.00 per person and year in the deworming area, are considerably higher than the costs of rabies immunisation, because of the increased size of the fox population and the existence of town foxes, but are not as high as to make an application over a large area unfeasible. Anthelmintic baiting has the potential to prevent severe human suffering [11, 39]. Whether municipalities in the study region decided to participate in baiting or not had never depended on the cost, but on public attitudes and the priorities set by political decision-makers (see also [22]).

If the One Health initiative [40] is to be implemented successfully, public health specialists, veterinarians and wildlife biologists should be involved in the projects.

## Supporting information

S1 Data (XLSX)
